# Increase in consumption of alcohol-based hand rub in German acute care hospitals over a 12 year period

**DOI:** 10.1186/s12879-021-06427-7

**Published:** 2021-08-06

**Authors:** Tobias Siegfried Kramer, Janine Walter, Christin Schröder, Michael Behnke, Jörg Clausmeyer, Christiane Reichardt, Petra Gastmeier, Karin Bunte

**Affiliations:** 1 Institute for Hygiene and Environmental Medicine Charité- Universitätsmedizin Berlin, Berlin, Germany; 2 National Reference Center for Surveillance of Nosocomial Infections, Hindenburgdamm 27 in, 12203 Berlin, Germany; 3 Aktion Saubere Hände Charité Universitätsmedizin Berlin, Berlin, Germany

**Keywords:** Hand hygiene; alcohol-based hand rub consumption, Compliance, Aktion Saubere Hände, Campaign, Patient safety; surveillance; Germany

## Abstract

**Background:**

Hand hygiene plays a crucial role in the transmission of pathogens and the prevention of healthcare-associated infections. In 2007, a voluntary national electronic surveillance tool for the documentation of consumption of alcohol-based hand rub (AHC) was introduced as a surrogate for hand hygiene compliance (HAND-KISS) and for the provision of benchmark data as feedback.

The aim of the study was to determine the trend in alcohol-based hand rub consumption between 2007 and 2018.

**Materials and methods:**

In this cohort study, AHC and patient days (PD) were documented on every ward in participating hospitals by trained local staff. Data was collected and validated in HAND-KISS. Intensive care units (ICU), intermediate care units (IMC), and regular wards (RW) that provided data during the study period between 2007 until 2018 were included into the study.

**Results:**

In 2018, 75.2% of acute care hospitals in Germany (*n* = 1.460) participated. On ICUs (*n* = 1998) mean AHC increased 1.74 fold (95%CI 1.71, 1.76; *p* < .0001) from 79.2 ml/PD to 137.4 ml/PD. On IMCs (*n* = 475) AHC increased 1.69 fold (95%CI 1.60, 1.79; *p* < .0001) from 41.4 ml/PD to 70.6 ml /PD..On RWs (*n* = 14,857) AHC was 19.0 ml/PD in 2007 and increased 1.71 fold (95%CI 1.70, 1.73; *p* < .0001) to 32.6 ml/PD in 2018.

**Conclusions:**

AHC in German hospitals increased on all types of wards during the past 12 years. Surveillance of AHC is widely established in German hospitals. Large differences among medical specialties exist and warrant further investigation.

## Background

Hand hygiene is one of the most effective measures for preventing healthcare-associated infections and the transmission of multidrug-resistant organisms [1, 2]. Adherence to the 5 Moments of Hand Hygiene remains of key importance for patient safety. However, reported compliance varies greatly [3]. Despite technical innovations and relevant limitations, direct observation based on the WHO protocol remains the gold standard [4]. Directly observed compliance rates are prone to the Hawthorne effect, overestimating performance [5]. Especially in overt observations improved compliance with hand hygiene can be occur in health care workers [6, 7]. In addition, this approach is time and resource intensive and might be difficult to implement in some centers [8]. Therefore, additional methods have proved necessary and have since been established. Consumption of alcohol-based hand rub (AHC) is used as a surrogate parameter for frequency of hand hygiene in various healthcare systems [9]. Application and interpretation of data can be difficult and has some pitfalls. The validity of AHC as a proxy for compliance with the 5 Moments model has not been conclusively established despite promising results [10]. While some reports describe a correlation with compliance on non-ICU inpatient wards [11, 12], others find no correlation between AHC and directly observed compliance [10]. Nonetheless, it provides opportunities for benchmarking and for additional feedback which is a key strategy for the improvement of hand hygiene compliance [13, 14].

In 2008 the national campaign for the improvement of hand hygiene in health care facilities (`Aktion Saubere Hände`) was launched in Germany. The campaign is ongoing. After a validation period, a surveillance module for alcohol-based hand rub consumption (AHC) was introduced in 2007 [15]. Documentation of consumption of alcohol-based hand rub on the ward level remained one of the mandatory elements for participation in the campaign [16] and is recommended in the official national hand hygiene guidelines for healthcare settings [17]. The number of participating hospitals has increased year by year. We previously reported an increase in AHC among wards that reported continuously every year between 2007 and 2015 [16].

The objective of this study was to show development of alcohol-based hand rub consumption in the period from 2007 until 2018in all wards and institutions that reported their rates of consumption.

## Materials and methods

The data presented in this study was derived from the voluntary German surveillance system for hand hygiene in health care institutions (HAND-KISS). Data on AHC and patient days (PD) was recorded by trained and designated infection prevention and control staff and submitted on an annual basis for every ward in each participating hospital. Data was aggregated based on HAND-KISS protocols and stratified into intensive care units (ICU), intermediate care units (IMC) and regular wards (RW) as well as by medical specialty, such as medical, surgical, pediatric and others [15, 16]. Specific information on the methodology of HAND-KISS and the calculation of reference data has been described in detail previously [16, 18]. All wards that provided data during the study period were included. Wards that provided annual data on AHC continuously during the study period 2007–2018 were included as the core group (CG) in this analysis.

AHC was calculated in milliliters (mL) per PD. The statistical significance of a change in AHC was determined using the Wilcox sign rank sum test. A *p*-value of <.05 was considered significant. Furthermore, AHC of ICU-CGs and RW-CGs were grouped separately into quartiles (Q) of baseline consumption in 2007 (Q1 ≤ 25%; Q2,> 25 to ≤50%; Q3, > 50 to ≤75%; Q4 > 75) in order to describe differences in their development.

The data utilised for this publication were anonymised or anonymized and collected in alignment with paragraph 23 the German Protection against Infection Act (“Infektionsschutzgesetz”). According this hospitals are required to routinely collect data on HAIs and aspects of HAI-prevention. Additional ethical approval and informed consent were therefore not required.

## Results

In 2018, a total 1460 hospitals reported AHC in 17,330 wards (14,857 RW, 1998 ICU and 475 IMC). A total 108 of 181 hospitals that participated in 2007 still had wards participating in CG and reporting annual data on AHC in 2018 (Table 1).

**Table 1:** Description of participating wards providing data in 2018

	total		ICU		IMC		RW	
	All n=	Core group n=	All n=	Core group n=	All n=	Core group n=	All n=	Core group n=
Hospitals	1460	108	1083	82	321	16	1448	99
Wards	17,330	912	1998	134	475	18	14,857	760
surgical	2846	165	224	15	39	1	2583	149
interdisciplinary	3075	138	1018	64	184	9	1873	65
medical	3583	207	255	20	68	1	3260	186
neonatology	255	21	186	16	16	1	53	4
pediatric	760	58	82	8	13	0	665	50
rehabilitation	488	3	15	0	4	0	469	3
Other non-surgical	3204	135	111	3	97	4	2996	128
Other surgical	3075	184	105	8	53	2	2917	174
Undefined	44	1	2	0	1	0	41	1
patientdays	866,974,407	82,559,533	49,144,866	6,432,414	9,549,448	765,479	808,280,093	75,361,640
Annual consumption of AHC (liters)	24,386,016	2,747,782	5,535,140	732,170	579,661	34,887	18,271,214	1,980,725

*ICU* Intensive care unit, *IMC* intermediate care unit, *RW* regular ward, *AHC* alcohol-based hand rub consumption

Annual data on AHC which was included in the analysis covered 16,213,364 PD in 2007 and covered 77,325,205 PD in 2018 (Table 2).

**Table 2:** Change of annual alcohol-based hand rub consumption (AHC) 2007–2018

						Distribution of AHC consumption ml/patient day							
Ward		Patient days		Annual consumption of AHC (liters)		MW		P25		P50		P75	
		All n=	Core n=	All n=	Core n=	All n=	Core n=	All n=	Core n=	All n=	Core n=	All n=	Core n=
ICU	2007	1,195,921	518,160	92,208	40,394	79.2	76.8	52.5	53.3	70.6	71.9	97.74	92.9
	2018	4,361,185	536,980	594,276	79,602	137.4	147.0	102.5	117.6	129.4	137.4	160.9	171.3
	Change [95%CI]				1.97 [1.96, 1.98]	1.74 [1.71, 1.76	1.92 [1.87, 1.96]						
	*p*-value					< 0.0001	< 0.0001						
IMC	2007	125,599	68,745	5195	1666	41.4	24.2	16.1	14.7	39.6	24.2	52.8	40.7
	2018	1,172,856	55,878	82,854	3771	70.6	67.5	45.3	54.6	64.0	59.2	93.0	82.7
	Change [95%CI]				2.26 [2.19, 2.34]	1.69 [1.60, 1.79]	2.18 [1.98, 2.41]						
	*p*-value					< 0.0001	0.0003						
RW	2007	14,891,844	6,363,820	243,159	101,957	19.0	18.3	10.2	10.2	14.5	14.3	20.6	20.3
	2018	71,791,164	5,983,841	2,112,330	211,363	32.6	38.4	22.0	26.7	28.8	33.5	37.8	43.4
	Change [95%CI]			8.69	2.07 [2.06, 2.08]	1.71 [1.70, 1.73]	2.10 [2.06, 2.14]						
	*p*-value					< 0.0001	< 0.0001						

Mann Whitney U test (overall); Wilcox sign rank sum test (core group); *ICU* Intensive care unit, *IMC* intermediate care unit, *RW* regular ward, *AHC* alcohol-based hand rub consumption

**Fig. 1:**
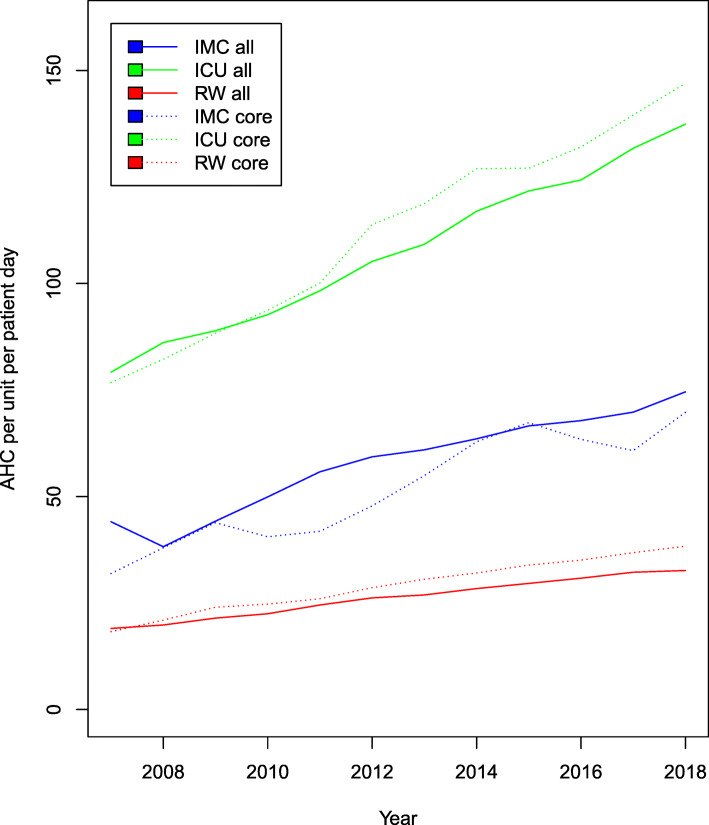
Development of alcohol-based hand rub consumption (AHC) in ml/PD 2007–2018 in the individual types of wards of all participants (all) and the core group (core). ICU: Intensive care unit, IMC: intermediate care unit, RW: regular ward, AHC: alcohol-based hand rub consumption

Significant increases in AHC were reported during the study period. However, differences in the development were observed in different types of wards (Fig. 1).

Median AHC increased from 70.6 ml/PD (IQR: 52.5–97.74) to 129.4 ml/PD (IQR: 102.5–160.9) on ICUs (1.74 fold; 95%CI 1.71, 1.76; *p* < .0001). Median AHC increased on IMCs from 39.6 ml/PD (IQR: 16.1–52.8) to 64.0 ml /PD (IQR: 45.3–93.0) (1.69 fold; 95%CI 1.60, 1.79; *p* < .0001). On regular wards median AHC was 14.45 ml/PD (IQR: 10.2.-20.6) in 2007 and increased to 28.8 ml/PD (IQR: 22.0–33.5) in 2018 (1.71 fold; 95%CI 1.70, 1.73; p < .0001). This development also applied to all types of wards in the core group (GC). ICU-CGs and RW-CGs had higher median AHC in 2018 compared to total participating ICUs and RWs.

**Fig. 2:**
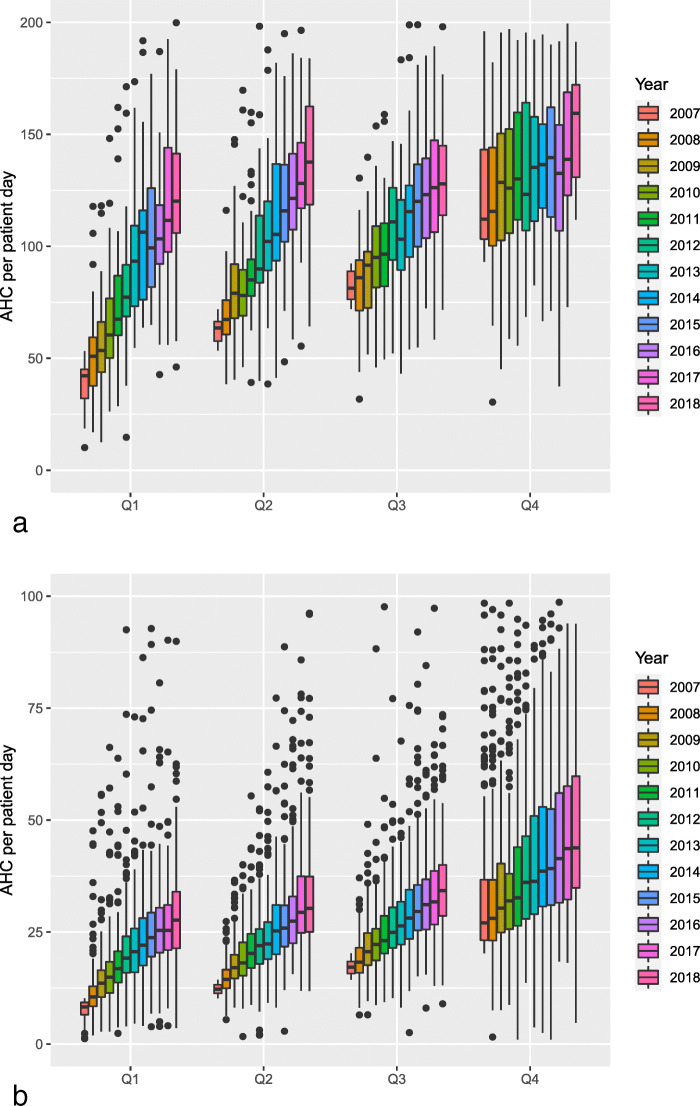
Development of annual alcohol-based hand rub consumption (AHC) in ml/PD 2007–2018 in core group dependant on quartile of the initial consumption in Intensive care units (**a**) and regular wards (**b**). Q: quartile

In addition, ICU-CGs with AHC in the lowest quartile in 2007 had the largest increase among ICUs, while it was slightly lower for the wards in the second and third quartiles (Fig. 2a).

Participating RW-CGs that began AHC in the lowest quartile had the largest increase over time. This also applied to wards in the second, third and fourth quartiles (Fig. 2b).

In 2018 wards caring for adult patients had lower AHC than wards caring for pediatric or neonatal patient populations (Table 3). Overall, median AHC was 129 ml/PD (IQR: 103–161) on ICUs while median AHC was 162 ml/PD (IQR: 129–216) on neonatal ICUs. Median AHC in pediatric wards on RWs was 30 ml/PD higher then overall median AHC on RWs.

**Table 3:** Distribution of alcohol-based hand rub consumption (AHC) according to type of ward and specialty

			Distribution of AHC consumption ml/patient day			
Ward	Patient days	Annual consumption of AHC (liters)	mean	25th percentile	median	75th percentile
**ICU**						
total	4,361,185	594,276	136	103	129	161
surgical	550,586	83,332	151	119	144	176
interdisciplinary	2,244,321	281,303	125	98	124	147
medical	486,144	65,277	134	103	126	162
neonatology	415,916	73,178	176	129	162	216
pediatric	146,714	24,788	169	130	164	213
rehabilitation	32,561	3471	107	103	111	120
**IMC**						
total	1,172,856	82,854	71	45	64	93
surgical	82,205	5983	73	48	62	89
interdisciplinary	500,670	36,218	72	48	69	95
medical	186,789	12,321	66	46	59	89
neonatology	30,700	2896	94	72	88	133
pediatric	15,860	2340	148	44	160	187
rehabilitation	12,740	748	59	53	62	71
**RW**						
total	71,792,092	2,112,330	29	22	29	38
surgical	12,840,675	379,371	30	22	28	35
interdisciplinary	9,154,385	262,671	29	23	29	37
medical	16,671,719	493,922	30	23	29	37
neonatology	117,162	10,318	88	53	77	114
pediatric	1,905,292	118,762	62	44	59	80
rehabilitation	4,187,022	63,967	15	11	19	28

*ICU* Intensive care unit, *IMC* intermediate care unit, *RW* regular ward, *AHC* alcohol-based hand rub consumption

## Discussion

Of 1942 acute care hospitals in Germany [19], 1460 provided data on AHC (75.2%). Between 2007 and 2018 median AHC increased almost two fold. We previously reported a similar increase in our CG [16]. However, the following results expand on this observation and cover all participating hospitals as well as wards in addition to the CG.

A recent point prevalence study found that median AHC was 20.3 ml/PD in European acute care hospitals (personal communication C. Suetens). This study also reported that ICUs in Europe had median AHC of 60 ml/PD. In this study the representative sample of participating German acute care hospitals (*n* = 46) AHC was slightly above the median (20.0–29.9 ml/PD). However, median AHC in all 218 German hospitals that participated in this point prevalence study was 35 ml/PD [20].

Reported AHC data on ward level from other campaigns or surveillance systems is scarce. Documentation and reporting of AHC is performed frequently in French hospitals. The mandatory ICSHA system documented an increase in median AHC from 6.1 ml/PD in 2006 [21] to an expected minimal consumption of 129 ml/PD on ICUs and 30 ml/PD on regular medical wards [22]. These results are similar to those observed in our voluntary system.

In order to establish reliable benchmarks, results need to be comparable [16]. Invasive treatment in ICU settings probably presents additional opportunities for hand hygiene which would result in higher AHC [18]. For this reason, participating wards have been stratified into ICU, IMC and RW, and further subdivided by medical specialty enabling valid assessment of the individual ward by the local IPC-team. The need for this stratification is especially apparent when comparing neonatal and pediatric ICUs with adult ICUs. Not only higher AHC, but also higher rates of compliance were reported in these wards. The underlying reasons are not yet fully understood.

The cause of the increase in AHC in German hospitals is probably multifactorial. Between 2008 until 2018, the national hand-hygiene campaign ‘Aktion-Saubere Hände’ launched annual initiatives focused on various topics related to hand hygiene. They provided a stream of new teaching and educational materials, successfully introduced a system for a certification procedure, and established direct observation [23]. These were adapted by many participating institutions and raised public awareness, which might also have had an influence. The increase in AHC in our cohort was independent of the type of ward but it was especially evident in RWs. The positive development was observed most prominently but not exclusively in CG wards. AHC increased more over time, especially on wards which had lower AHC in 2007. These findings suggest the effectiveness of a long-term campaign on hand hygiene when combined with a voluntary surveillance system and the benefit of benchmarking and feedback by local infection prevention and control staff.

Several limitations apply to this study. Due to the voluntary nature of our surveillance system, it is possible that only highly motivated institutions participated. Continuous documentation of AHC, annual interpretation of results, and the implementation of measures for improvement are mandatory legal requirements for healthcare institutions in Germany. A majority of hospitals in Germany have opted to use our surveillance system. Therefore, selection processes appear less important for the external validity of the data.

It is possible that changing features of the patient populations and changes in the structure of hospitals had an influence on AHC. Nevertheless, the increase in AHC was seen in wards in CGs that did not change over time.

The correlation of AHC and compliance is not fully clear. Therefore, AHC can only be a surrogate of compliance to the 5 Moments of Hand Hygiene on ward level. It is possible that uptake and availability of AHC improved but that compliance did not. An increase in AHC and, therefore, also of hand disinfections performed per patient-day only reflect a fraction of the hand hygiene opportunities occurring on wards [24]. The nearly two fold increase in AHC is certainly not associated with a two-fold increase in compliance [23]. Direct overt observation of hand hygiene compliance provides only a little insight into daily practice, which is subject to uncertainties [25]. Documentation of AHC/PD allows for an easy and cost effective approach for a surrogate, providing a context for the results of direct observation.

## Conclusions

Consumption of alcohol-based hand rub increased between 2007 and 2018 on every type of ward that participated. The increase in AHC was even higher in wards that started in the lower quartiles of distribution. Surveillance of AHC is now widely established in German hospitals as a tool for providing feedback. Further research is necessary to determine the underlying reasons for differences among specialties and the efficacy of feedback on AHC on the quality of hand hygiene as well as on compliance with the WHO 5 Moments of Hand Hygiene.

## Data Availability

The datasets used and/or analyzed during the current study are available from the corresponding author on reasonable request.

## Abbreviations

95% CI: 95% Confidence interval
AHC: Alcohol-based handrub consumption
ASH: Aktion saubere hände (national hand hygiene campaign)
CG: Core group
HAND-KISS: National hospital hand hygiene surveillance system
ICU: Intensive care unit
IMC: Intermediate care unit
IQR: Interquartile range
ml: Milliliters
OR: Odds ratio
PD: Patient days
Q: Quartile
RW: Regular ward
